# Differentiation of Neoplastic and Non-neoplastic Intracranial Enhancement Lesions Using Three-Dimensional Pseudo-Continuous Arterial Spin Labeling

**DOI:** 10.3389/fnins.2022.812997

**Published:** 2022-02-24

**Authors:** Wen-zhong Hu, Fan Guo, Yong-qiang Xu, Yi-bin Xi, Bei He, Hong Yin, Xiao-wei Kang

**Affiliations:** ^1^Department of Radiology, Xi’an People’s Hospital, Xi’an Fourth Hospital, Xi’an, China; ^2^Department of Radiology, Xijing Hospital, The Fourth Military Medical University, Xi’an, China

**Keywords:** three-dimensional pseudo-continuous arterial spin labeling, non-neoplastic, high-grade gliomas, metastasis, cerebral blood flow

## Abstract

**Background and Purpose:**

It is sometimes difficult to effectively distinguish non-neoplastic from neoplastic intracranial enhancement lesions using conventional magnetic resonance imaging (MRI). This study aimed to evaluate the diagnostic performance of three-dimensional pseudo-continuous arterial spin labeling (3D-pCASL) to differentiate non-neoplastic from neoplastic enhancement lesions intracranially.

**Materials and Methods:**

This prospective study included thirty-five patients with high-grade gliomas (HGG), twelve patients with brain metastasis, and fifteen non-neoplastic patients who underwent conventional, contrast enhancement and 3D-pCASL imaging at 3.0-T MR; all lesions were significantly enhanced. Quantitative parameters including cerebral blood flow (CBF) and relative cerebral blood flow (rCBF) were compared between neoplastic and non-neoplastic using Student’s *t*-test. In addition, the area under the receiver operating characteristic (ROC) curve (AUC) was measured to assess the differentiation diagnostic performance of each parameter.

**Results:**

The non-neoplastic group demonstrated significantly lower rCBF values of lesions and perilesional edema compared with the neoplastic group. For the ROC analysis, both relative cerebral blood flow of lesion (rCBF-L) and relative cerebral blood flow of perilesional edema (rCBF-PE) had good diagnostic performance for discriminating non-neoplastic from neoplastic lesions, with an AUC of 0.994 and 0.846, respectively.

**Conclusion:**

3D-pCASL may contribute to differentiation of non-neoplastic from neoplastic lesions.

## Introduction

The incidence of brain and central nervous system tumors has been increasing in recent years. There are about 308,000 new cases of brain tumors worldwide until 2020. The morbidity rates of men and women are 3.9% and 3.0%, respectively, and the mortality rates are 3.2% and 2.4%, respectively (Sung et al., 2021). Conventional MRI has a good value in differentiating diagnosis by assessing the shape, location, and mass effect of lesions (Villanueva-Meyer et al., 2017). Sometimes, conventional MRI of many intracranial non-neoplastic diseases, such as brain abscess, tuberculosis, and cerebral cysticercosis, mimics cerebral high-grade glioma, which could all manifest as space-occupying lesions with cystic, necrosis, and ring enhancement (Omuro et al., 2006). However, the treatment strategies and prognosis of non-neoplastic and neoplastic are completely different, so how to accurately differentiate those two kinds of diseases is quite important (Bush et al., 2017).

Considering the limitations of conventional MRI, a growing number of studies have focused on assessing the physiological and metabolic characteristics of lesions, especially on tumors (Ko et al., 2016). Arterial spin labeling (ASL) is an emerging MRI perfusion technology that uses magnetically labeled arterial blood water protons as an endogenous tracer. ASL can quantitatively measure the relative cerebral blood flow (rCBF) in different lesions and evaluate the malignant degree of lesions from the level of microcirculation (Hernandez-Garcia et al., 2019). According to different labeling methods, ASL can be divided into three types: pulsed ASL (PASL), continuous ASL (CASL), and pseudo-continuous ASL (pCASL). pCASL has advantages including high signal-to-noise ratio, high labeling efficiency, minimal magnetization transfer effect, less radiofrequency energy deposition, low requirements for hardware equipment, and good repeatability (Wu et al., 2014; Alsop et al., 2015). 3D-pCASL is a more advanced technology that can improve the image signal-to-noise ratio further and reduce motion artifacts while achieving whole brain volume perfusion imaging.

Previous studies have proved that ASL is valuable in identifying and grading brain tumors (Haller et al., 2016; Abdel Razek et al., 2019b; Xi et al., 2019). As far as we know, there are few studies regarding using ASL to distinguish intracranial non-neoplastic from neoplastic lesions. In this retrospective study, we collected imaging data of patients with intracranial non-neoplastic and neoplastic lesions and analyzed the difference between them on ASL images. This study aimed to evaluate the additional value of ASL in distinguishing non-neoplastic from neoplastic lesions in intracranial, which manifest as ring-enhancement lesions.

## Materials and Methods

### Subjects

This retrospective study was approved by the Institutional Review Board, and informed consent was obtained from all participants. From September 2015 to September 2020, MRI data of patients from our hospital were retrospectively reviewed. The inclusion criteria were as follows: (1) all patients were newly diagnosed, untreated patients; (2) all patients underwent conventional MRI including T1, T2, post-contrast T1 weighted imaging, and 3D-pCASL preoperatively; (3) all lesions showed obvious ring enhancement from the contrast MRI scanning; and (4) all patients were further confirmed by pathological or clinical examinations. The following were the exclusion criteria: (1) poor image quality, which affects the measurement results, and (2) those who had received therapy prior to MRI. Finally, 35 HGG (21 men, 14 women, mean age 52.9 years, range 12–79 years), 12 metastases (7 men, 5 women, mean age 57.3 years, range 42–77 years), and 15 non-neoplastic cases (9 men, 6 women, mean age 39 years, range 10–64 years) were enrolled. The non-neoplastic lesions contained the following: brain abscess (*n* = 7), tuberculoma (*n* = 3), granuloma (*n* = 3), cerebral cysticercosis, and demyelinating pseudotumor (*n* = 1). Among the 12 patients with brain metastases, the primary sites of tumors were lung cancer (*n* = 5), stomach cancer, osteosarcoma, cervical cancer, parotid gland cancer, colon cancer, rectal cancer, and adenocarcinoma (*n* = 1 each).

### Magnetic Resonance Imaging Acquisition

In this experiment, a 3.0-T MR system (Discovery MR750, GE Healthcare, Milwaukee, WI, United States) with an 8-channel head-matrix coil was used. The head of the patient was fixed with a sponge cushion, and the patient was supine on the examination table. The scan sequences included the following: (1) axial T1WI [TR, 1,750 ms; TE, 24 ms; section thickness, 4 mm; inter-slice gap = 0 mm; field of view (FOV), 240 × 240 mm^2^; matrix, 320 × 256]; (2) T2WI (TR, 3,976 ms; TE, 92 ms; section thickness, 5 mm; inter-slice gap = 1.5 mm; FOV, 240 × 240 mm^2^; matrix size, 512 × 512); (3) fluid-attenuated inversion recovery (FLAIR) (TR, 8,400 ms; TE, 145 ms; section thickness, 5 mm; inter-slice gap = 1.5 mm; FOV, 240 × 240 mm^2^; matrix size, 160 × 256); (4) post-contrast T1 was acquired after intravenous administration of 0.1 mmol/kg gadopentetate dimeglumine; (5) ASL was performed with pseudocontinuous labeling, background suppression, and a stack of spiral 3D fast spin-echo imaging sequences using the following acquisition parameters: 512 sampling points on eight spirals, TR = 4,632 ms; TE = 10.5 ms; matrix = 128 × 128; slice thickness = 4 mm, field of view (FOV) = 240 × 240 mm^2^; scan time = 4 min 27 s; post-labeling delay = 1 m, 525 ms.

### Image Processing and Analysis

The image data were transferred to an offline workstation for post-processing (Advantage Workstation, AW4.5; GE Medical Systems) provided by the supplier, and the CBF images were post-processed by GE FuncTool software. We used T1WI enhancement and FLAIR to define the localization of the lesions and perilesional edema, and then registered these images with 3D-pCASL images. Cerebral blood flow (CBF) values were measured by placing the regions of interest (ROI) above the lesion with the highest perfusion signal. CBF of the solid part of the lesion (CBF-L), CBF of the PLE (CBF-PLE) (within 1 cm from the lesion), and CBF of contralateral normal gray matter (CBF-CGM) were measured, respectively. CBF-PLE was measured by placing the ROI above the highest perfusion signal seen in the perilesional edema on ASL map. Two experienced neuroradiologists who were blinded to the final diagnosis measured 3 times and then taken the average value. The necrosis, cystic change, hemorrhage, or blood vessel area that may affect the measurement were avoided. To minimize the inter-individual variation in CBF values, the rCBF values (rCBF-L, rCBF-PLE) were calculated by normalizing to the CBF-CGM.

### Statistical Analysis

All data were processed by SPSS 23.0 software, and all values were expressed as mean ± standard deviation (SD). Interobserver agreement for the CBF values measured by the two observers was analyzed by calculating the intraclass correlation coefficient (ICC). ICCs greater than 0.74 were considered to be excellent (Yeganeh et al., 2019). Two observers averaged the measured values of each patient for further analysis. The comparison of measurement data that conform to the normal distribution used the independent sample *t*-test, and qualitative data used the chi-square test. The area under the curve (AUC) from receiver operating characteristic (ROC) analysis was used to evaluate the diagnostic performance of the ASL-determined rCBF for differentiating non-neoplastic from neoplastic lesions. *p* values < 0.05 were considered to be statistically significant.

## Results

### Patient Demographics and Conventional Magnetic Resonance Imaging Features

**FIGURE 1:**
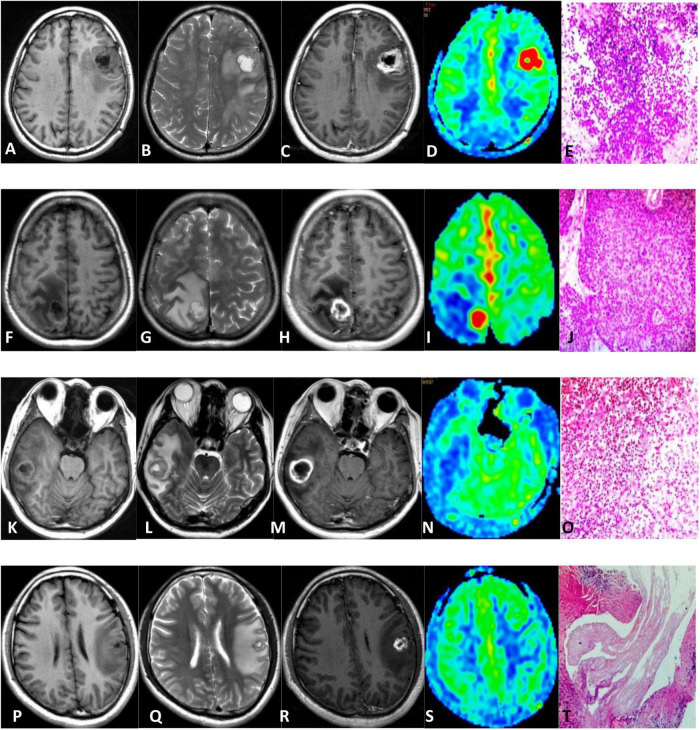
Comparison of HGG **(A–E)**, metastasis **(F–J)**, brain abscess **(K–O)**, and cerebral cysticercosis **(P–T)**. First row: 64-year-old male with an HGG in the left frontal lobe; unenhanced MR scan showed hypointense on T1WI and hyperintense onT2WI **(A,B)**. The contrast-enhanced T1-weighted MRI showed ring enhancement **(C)**. 3D-pCASL exhibited as hyper-perfusion in the region of tumor compared with the contralateral brain **(D)**. Pathologically, the tumor cells were diffusely distributed and showed increased density **(E)**. Second row: 42-year-old woman with a metastatic tumor in the right parietal lobe; MR plain scan showed hypointense on T1WI and hyperintense on T2WI **(F,G)**. The enhanced scan showed ring enhancement **(H)**. 3D-pCASL showed as hyper-perfusion in the region of metastasis **(I)**. Pathology showed the nested atypical cells, which was squamous cell carcinoma **(J)**. Third row: 40-year-old man with a brain abscess in the right temporal lobe. MR plain scan showed hypointense on T1WI and hyperintense on T2WI **(K,L)**. The enhanced scan showed ring enhancement **(M)**. 3D-pCASL showed the lesion demonstrated as hypo-perfusion **(N)**. Pathology showed lymphocyte, neutrophil infiltration, abscess formation, and granulation tissue hyperplasia **(O)**. Fourth row: 28-year-old man with cerebral cysticercosis in the left frontal and parietal lobe. MR plain scan showed hypointense on T1WI and hyperintense on T2WI **(P,Q)**. The enhanced scan showed ring enhancement **(R)**. 3D-pCASL showed that the lesion was iso-hypo-perfusion **(S)**. Pathologically, lace-like parasites can be seen **(T)**.

The clinical and MRI characteristics of subjects are summarized in Table 1. There was no statistical difference in male-to-female ratio between the two groups. The age of the non-neoplastic group was younger than the neoplastic group with statistically significant difference. All intracranial lesions were exhibited as ring-like enhancement and accompanied with perilesional edema. The representative MRI of a patient with HGG, metastasis, and non-neoplastic lesions is shown in Figure 1.

**TABLE 1:** Clinical and MRI characteristics of patients.

	Neoplastic (*n* = 47)	Non-neoplastic (*n* = 15)	*p*-values
Age (years)	54.09 ± 12.55	39.00 ± 17.03	<0.001
Sex (male:female)	28:19	9:6	0.977
Number of lesions			
Single lesion	37	11	-
Multiple lesions	10	4	-
Enhancing pattern	Ring enhancement	Ring enhancement	-
Necrosis	47	15	-

*Values are expressed as mean ± standard deviation (SD).*

*The Student’s t-test was used to compare the age between the two groups, and the chi-square test was used to compare the gender.*

### Differences in Cerebral Blood Flow and Relative Cerebral Blood Flow Between Neoplastic and Non-neoplastic Groups

Supplementary Table 1 shows the ICCs measured by two observers. Excellent agreement was observed for CBF values. The CBF and rCBF values of the lesions and perilesional edema in the non-neoplastic group were significantly lower than those in the neoplastic group. There was no significant difference in the CBF-CGM values between the two groups, as shown in Table 2. The representative ASL images are shown in Figure 1.

**TABLE 2:** 3D-pCASL imaging-derived parameters for non-neoplastic and neoplastic.

	Neoplastic (*n* = 47)	Non-neoplastic (*n* = 15)	*p*-values
CBF-L	115.59 ± 40.83	40.17 ± 6.71	<0.001
CBF-PLE	29.80 ± 6.29	22.22 ± 2.29	<0.001
CBF-CGM	54.05 ± 2.81	53.50 ± 3.13	0.521
rCBF-L	2.13 ± 0.75	0.75 ± 0.14	<0.001
rCBF-PLE	0.55 ± 0.12	0.42 ± 0.04	<0.001

*Values are expressed as mean ± standard deviation (SD).*

*Comparisons between groups were performed by Student’s t-test.*

*CBF-L, cerebral blood flow of lesion; CBF-PLE, cerebral blood flow of perilesional edema; CBF-CGM, cerebral blood flow of contralateral normal gray matter; rCBF-L, relative cerebral blood flow of lesion; rCBF-PLE, relative cerebral blood flow of perilesional edema.*

After comparing the neoplastic group and the non-neoplastic group, the CBF-L, CBF-PLE, rCBF-L, and rCBF-PLE values of the metastatic group and the non-neoplastic group were lower than those of the HGG group, and the difference was statistically significant (*p* < 0.001). The CBF-L and rCBF-L values of the non-neoplastic group were lower than those of the metastatic group, and the difference was statistically significant (*p* < 0.001), while the CBF-PLE and rCBF-PLE were not significantly different between the two groups with *p*-values of 0.894 and 0.795, respectively. The details are shown in Supplementary Table 2.

### Diagnostic Values of Relative Cerebral Blood Flow of Lesion and Relative Cerebral Blood Flow of Perilesional Edema

**FIGURE 2:**
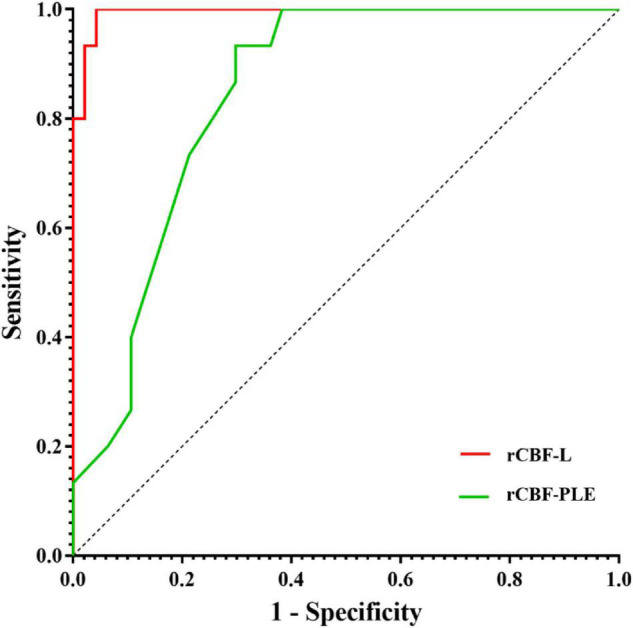
Receiver operating characteristic curves for rCBF and rCBF-PLE for differentiating non-neoplastic from neoplastic lesions. AUC, area under the receiver operating characteristic curve; rCBF-L, relative cerebral blood flow of lesion; rCBF-PLE, relative cerebral blood flow of perilesional edema.

Receiver operating characteristic analysis showed that the AUC of rCBF-L for the diagnosis of non-neoplastic and neoplastic was 0.994 with a sensitivity of 95.7% and a specificity of 100%. The AUC of rCBF-PLE for the diagnosis of non-neoplastic and neoplastic was 0.846 with a sensitivity of 70.2% and a specificity of 93.3%. Table 3 summarizes the best cutoff values for different parameters that distinguish non-neoplastic from neoplastic lesions. The ROC curves are shown in Figure 2.

**TABLE 3:** Diagnostic performance of rCBF-L and rCBF-PLE for differentiating non-neoplastic from neoplastic lesions.

Model	AUC	Cutoff value	Sensitivity	Specificity
rCBF-L	0.994 (0.983–1.000)	1.095	0.957	1.000
rCBF-PLE	0.846 (0.752–0.940)	0.475	0.702	0.933

*Data in parentheses are 95% confidence intervals.*

*AUC, area under the receiver operating characteristic curve; rCBF-L, relative cerebral blood flow of lesion; rCBF-PLE, relative cerebral blood flow of perilesional edema.*

After the main group comparison, we found that when the rCBF-L cutoff value was selected at 1.545, the AUC for the diagnosis of HGG and metastasis was 0.923 (95% CI 0.839–1.000), with a sensitivity of 91.4% and a specificity of 83.3%. When the rCBF-L cutoff value was selected at 0.940, the AUC for diagnosing metastatic and non-neoplastic lesions was 0.978 (95% CI 0.934–1.000), with a sensitivity of 91.7% and a specificity of 93.3%. When the rCBF-PLE cutoff value was selected at 0.465, the AUC for the diagnosis of HGG and metastases was 0.923 (95% CI 0.949–1.000), with a sensitivity of 94.3% and a specificity of 100%.

## Discussion

In the present study, we evaluated the utility of 3D-PCASL for the differentiation of neoplastic and non-neoplastic lesions. We demonstrated that non-neoplastic lesions exhibited lower CBF values based on ASL perfusion MRI compared with neoplastic lesions. We demonstrated that 3D-PCASL MRI techniques could be used for differentiation with high accuracy.

It is essential to distinguish non-neoplastic from neoplastic lesions as the therapeutic strategies and prognosis are different clinically. However, many non-neoplastic neurological diseases could often mimic brain tumors in neuroimaging (Okamoto et al., 2004a,b). The conventional MR contrast enhancement is affected by the damage of the blood–brain barrier (BBB) and the permeability of neovascularization. Most intracranial tumors could destroy the BBB, while some non-neoplastic lesions could increase vascular permeability (Gupta et al., 2008). Therefore, both intracranial neoplastic and non-neoplastic lesions could show significant enhancement, usually ring enhancement, and thus difficult to distinguish. Most brain abscesses show high signal on MR diffusion-weighted imaging (DWI), which is helpful to distinguish from the necrosis center of neoplastic disease. However, there are reports that some metastases and HGG could also show high signal on DWI (Holtås et al., 2000; Hakyemez et al., 2005). Tuberculoma and metastasis could both show lipid peaks on magnetic resonance spectroscopy (MRS); as a result, MRS may not be useful when calcification and bleeding exist (Nagar et al., 2007). Misdiagnosis may expose patients to unnecessary surgery. Accordingly, it is particularly important to find a new diagnostic method. All the above-mentioned sequences could not reflect the blood flow distribution and perfusion in the lesions.

In this study, we first focused on the application of 3D-pCASL in intracranial enhancement lesions. Previous examinations of CBF were mostly accomplished through perfusion imaging based on nuclear medicine, dynamic contrast-enhanced computed tomography (CT), or dynamic susceptibility contrast MRI (DSC-MRI) (Noguchi et al., 2016). However, nuclear medicine examinations cannot be routinely performed in clinical practice. Dynamic contrast-enhanced CT requires the use of contrast agents and gives radiation to the patient. DSC-MRI has shown good ability in differentiating non-neoplastic from neoplastic lesions and is widely used. Studies have shown that the relative cerebral blood volume (rCBV) of non-neoplastic lesions was significantly lower than that of neoplastic lesions (Floriano et al., 2013). However, DSC can be easily affected by the destruction of the BBB and requires the injection of contrast agents. ASL is one of the non-contrast-enhanced MR perfusion imaging methods, which has the advantages of non-invasiveness, simple operation, and repeatable inspection. It is widely used in the differential diagnosis between tumors and grading of gliomas (Suh et al., 2018). ASL can more realistically reflect the angiogenesis of the lesion without being affected by blood products, necrosis, or calcification (Furtner et al., 2014). Many previous studies have shown that there is a strong correlation between the perfusion parameters obtained by ASL and DSC (Grade et al., 2015; Soni et al., 2017; Novak et al., 2019), which showed the feasibility of ASL as an alternative to DSC.

In the current study, the CBF-L and rCBF-L of non-neoplastic lesions were lower compared with neoplastic lesions, which is consistent with previous studies (Soni et al., 2018). This result may be attributed to the fact that neoplastic lesions could induce angiogenesis, leading to an increase in blood perfusion. Muccio et al. (2008) found lower rCBV values in patients with brain abscesses, proposing that this was due to the low density of capillaries and the enrichment of collagen fibers in the capsule of abscess lesions. This explanation also applies to the performance of other non-neoplastic lesions with low perfusion. Therefore, when encountering a rare HGG with a high DWI signal in clinical practice (Reiche et al., 2010), ASL could be helpful for the distinguishing diagnosis from brain abscess. At the same time, we found that the CBF-L and rCBF-L of metastatic lesions were lower than HGG, which is also consistent with previous studies (Sunwoo et al., 2016; Abdel Razek et al., 2019a). This can be attributed to the higher density of new blood vessels in HGG.

Secondly, we tested the application of 3D-pCASL in perilesional perfusion. Compared with brain tumors, non-neoplastic lesion has lower CBF-PLE and rCBF-PLE. However, there was no significant difference in CBF PLE and rCBF PLE between metastatic and non-neoplastic lesions. This may be due to the tendency of glioma cells to infiltrate surrounding brain tissues. The PLE of glioma includes not only the invading tumor cells, but also the glial alterations in surrounding normal tissues, such as swelling of astrocytes, aggregation of microglia, and microglia activation (Engelhorn et al., 2009). The PLE of metastases represents pure vasogenic edema rich in plasma proteins, and its source is the leakage of capillaries in or around the metastasis. In addition, the decrease of CBF-PLE may be caused by local compression of the microcirculation by edema (Lin et al., 2016).

Our study has some limitations. First, there may be a bias of sample selection in retrospective studies. Second, the sample size was not large enough, and the data had a large degree of dispersion. More patients will validate our results in the future. Finally, it has been reported that factors such as MR sequence parameters and postprocessing software may influence the results.

## Conclusion

In this study, we found that both the rCBF value of the lesion and the peri-edema from the non-neoplastic group were significantly lower than those of the neoplastic group. ASL parameters could be helpful in discriminating non-neoplastic from neoplastic lesions for the intracranial enhancement lesions, thus preventing misdiagnosis and unnecessary surgery.

## Data Availability Statement

The original contributions presented in the study are included in the article/Supplementary Material, further inquiries can be directed to the corresponding author/s.

## Ethics Statement

Written informed consent was obtained from the individual(s), and minor(s)’ legal guardian/next of kin, for the publication of any potentially identifiable images or data included in this article.

## Author Contributions

HY, Y-BX, and X-WK contributed to conception and design of the study. W-ZH and Y-QX organized the database. Y-QX and FG performed the statistical analysis. W-ZH wrote the first draft of the manuscript. FG and BH wrote sections of the manuscript. All authors contributed to manuscript revision, read, and approved the submitted version.

## Conflict of Interest

The authors declare that the research was conducted in the absence of any commercial or financial relationships that could be construed as a potential conflict of interest.

## Publisher’s Note

All claims expressed in this article are solely those of the authors and do not necessarily represent those of their affiliated organizations, or those of the publisher, the editors and the reviewers. Any product that may be evaluated in this article, or claim that may be made by its manufacturer, is not guaranteed or endorsed by the publisher.
