# Supplementary material for: Differentiation of Neoplastic and Non-neoplastic Intracranial Enhancement Lesions Using Three-Dimensional Pseudo-Continuous Arterial Spin Labeling
Source: Front Neurosci. 2022 Feb 24;16:812997. doi: 10.3389/fnins.2022.812997 (PMC8923048; doi:10.3389/fnins.2022.812997)
Supplement: Supplementary file 1 [file Data_Sheet_1.docx]

**Supplementary Table 1**

Inter-observer agreement

|  | CBF-L | CBF-PLE | CBF-CGM |
| --- | --- | --- | --- |
| HGG | 0.992 | 0.812 | 0.764 |
| metastases | 0.993 | 0.806 | 0.797 |
| non-neoplastic | 0.903 | 0.817 | 0.806 |

ICC=intraclass correlation coefficient; HGG= high-grade gliomas; CBF-L= cerebral blood flow of lesion; CBF-PLE= cerebral blood flow of perilesional edema; CBF-CGM= cerebral blood flow of contralateral normal grey matter.

| **Supplementary Table 2**  3D-pCASL imaging-derived parameters for HGG, metastasis and non-neoplastic | | | | |
| --- | --- | --- | --- | --- |
|  | HGG  （n=35） | Metastasis  （n=12） | non-neoplastic  （n=15） | *P* values |
| CBF-L | 129.48±36.67 | 75.07±20.22 | 40.16±6.71 | ＜0.001,＜0.001,＜0.001 |
| CBF-PLE | 32.36±5.09 | 22.33±1.94 | 22.22±2.29 | ＜0.001,0.894,＜0.001 |
| rCBF-L | 2.39±0.67 | 1.38±0.36 | 0.75±0.14 | ＜0.001,＜0.001,＜0.001 |
| rCBF-PLE | 0.60±0.98 | 0.41±0.28 | 0.41±0.38 | ＜0.001,0.795,＜0.001 |
| Values are expressed as mean ± standard deviation (SD). The *P* values represented the comparison results of HGG with metastases, metastases with non-neoplastic, and HGG with non-neoplastic by student’s t-test. HGG= high-grade gliomas ;CBF-L= cerebral blood flow of lesion; CBF-PLE= cerebral blood flow of perilesional edema ; rCBF-L= relative cerebral blood flow of lesion; rCBF-PLE= relative cerebral blood flow of perilesional edema. | | | | |
